# Targeting macrophages with multifunctional nanoparticles to detect and prevent atherosclerotic cardiovascular disease

**DOI:** 10.1093/cvr/cvae099

**Published:** 2024-05-02

**Authors:** Victoria Nankivell, Achini K Vidanapathirana, Ayla Hoogendoorn, Joanne T M Tan, Johan Verjans, Peter J Psaltis, Mark R Hutchinson, Brant C Gibson, Yiqing Lu, Ewa Goldys, Gang Zheng, Christina A Bursill

**Affiliations:** Australian Research Council (ARC) Centre of Excellence for Nanoscale BioPhotonics (CNBP); Vascular Research Centre, Lifelong Health, South Australian Health and Medical Research Institute (SAHMRI), North Terrace, Adelaide, 5000, Australia; Faculty of Health and Medical Science, The University of Adelaide, North Terrace, Adelaide, 5000, Australia; Australian Research Council (ARC) Centre of Excellence for Nanoscale BioPhotonics (CNBP); Vascular Research Centre, Lifelong Health, South Australian Health and Medical Research Institute (SAHMRI), North Terrace, Adelaide, 5000, Australia; Faculty of Health and Medical Science, The University of Adelaide, North Terrace, Adelaide, 5000, Australia; Australian Research Council (ARC) Centre of Excellence for Nanoscale BioPhotonics (CNBP); Vascular Research Centre, Lifelong Health, South Australian Health and Medical Research Institute (SAHMRI), North Terrace, Adelaide, 5000, Australia; Vascular Research Centre, Lifelong Health, South Australian Health and Medical Research Institute (SAHMRI), North Terrace, Adelaide, 5000, Australia; Faculty of Health and Medical Science, The University of Adelaide, North Terrace, Adelaide, 5000, Australia; Australian Research Council (ARC) Centre of Excellence for Nanoscale BioPhotonics (CNBP); Vascular Research Centre, Lifelong Health, South Australian Health and Medical Research Institute (SAHMRI), North Terrace, Adelaide, 5000, Australia; Faculty of Health and Medical Science, The University of Adelaide, North Terrace, Adelaide, 5000, Australia; Australian Research Council (ARC) Centre of Excellence for Nanoscale BioPhotonics (CNBP); Vascular Research Centre, Lifelong Health, South Australian Health and Medical Research Institute (SAHMRI), North Terrace, Adelaide, 5000, Australia; Faculty of Health and Medical Science, The University of Adelaide, North Terrace, Adelaide, 5000, Australia; Australian Research Council (ARC) Centre of Excellence for Nanoscale BioPhotonics (CNBP); Faculty of Health and Medical Science, The University of Adelaide, North Terrace, Adelaide, 5000, Australia; Australian Research Council (ARC) Centre of Excellence for Nanoscale BioPhotonics (CNBP); School of Science, RMIT University, Melbourne, Victoria, Australia; Australian Research Council (ARC) Centre of Excellence for Nanoscale BioPhotonics (CNBP); School of Engineering, Macquarie University, Sydney, NSW, Australia; Australian Research Council (ARC) Centre of Excellence for Nanoscale BioPhotonics (CNBP); Graduate School of Biomedical Engineering, University of New South Wales, High Street, NSW, 2052, Australia; Australian Research Council (ARC) Centre of Excellence for Nanoscale BioPhotonics (CNBP); Department of Medical Biophysics, University of Toronto, 101 College Street, Toronto, M5G 1L7, Canada; Australian Research Council (ARC) Centre of Excellence for Nanoscale BioPhotonics (CNBP); Vascular Research Centre, Lifelong Health, South Australian Health and Medical Research Institute (SAHMRI), North Terrace, Adelaide, 5000, Australia; Faculty of Health and Medical Science, The University of Adelaide, North Terrace, Adelaide, 5000, Australia

**Keywords:** Nanoparticles, Atherosclerosis, Macrophages

## Abstract

Despite the emergence of novel diagnostic, pharmacological, interventional, and prevention strategies, atherosclerotic cardiovascular disease remains a significant cause of morbidity and mortality. Nanoparticle (NP)-based platforms encompass diverse imaging, delivery, and pharmacological properties that provide novel opportunities for refining diagnostic and therapeutic interventions for atherosclerosis at the cellular and molecular levels. Macrophages play a critical role in atherosclerosis and therefore represent an important disease-related diagnostic and therapeutic target, especially given their inherent ability for passive and active NP uptake. In this review, we discuss an array of inorganic, carbon-based, and lipid-based NPs that provide magnetic, radiographic, and fluorescent imaging capabilities for a range of highly promising research and clinical applications in atherosclerosis. We discuss the design of NPs that target a range of macrophage-related functions such as lipoprotein oxidation, cholesterol efflux, vascular inflammation, and defective efferocytosis. We also provide examples of NP systems that were developed for other pathologies such as cancer and highlight their potential for repurposing in cardiovascular disease. Finally, we discuss the current state of play and the future of theranostic NPs. Whilst this is not without its challenges, the array of multifunctional capabilities that are possible in NP design ensures they will be part of the next frontier of exciting new therapies that simultaneously improve the accuracy of plaque diagnosis and more effectively reduce atherosclerosis with limited side effects.

## Graphical Abstract

**Graphical Abstract:**
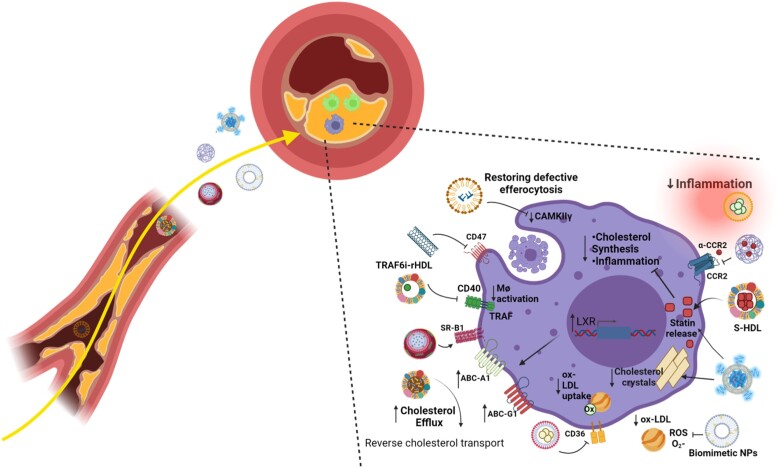

## 1. Introduction

Despite rapid progress in treatment, diagnosis, and advances in revascularization technologies for atherosclerotic cardiovascular disease (CVD), the current incidence of serious and recurrent cardiovascular events remains unacceptably high, even when patients are receiving optimal medical care.^1^ Overcoming this challenge requires a multi-step approach to (i) improve early disease detection, classification, and prevention and (ii) advance effective treatment strategies that improve risk factor management beyond cholesterol lowering.

One of the central problems in CVD diagnostics is the accurate detection and prediction of whether a plaque is or will become unstable.^2^ Current clinically available imaging techniques predominantly focus on detecting general plaque morphology and size, rather than the molecular and cellular composition of the plaque, despite these latter features being critically linked to future events.^3^ They are limited by inadequate sensitivity, specificity, and spatial resolution that may not provide early detection. Furthermore, imaging-guided assessments of luminal stenosis do not reliably relate to the risk of myocardial infarction (MI). Plaques at high risk of rupture may appear invisible by imaging because they reside in the artery wall.^4^ Similarly, current therapeutic strategies have their own limitations by not considering the biological complexities of atherosclerosis. Conventional lipid-lowering statin therapies do not target the array of mechanisms that drive plaque development. This may explain why an increasing and substantial proportion of patients with MI (15%) fall outside the traditional risk factor algorithm that mainly considers circulating factors.^5^ As a result, the lack of early detection and effective secondary treatment strategies present an urgent unmet clinical need for new imaging technologies and agents that add benefit on top of current lipid-lowering therapies to improve the management of atherosclerosis.

Nanoparticles (NPs) are formulations of diverse molecular compounds which are spatially organized on the scale of tens of nanometres. With often complex architecture, they offer a range of sophisticated functionalities capable of advancing both the diagnosis and treatment of atherosclerosis.^6^ Unlike conventional pharmacotherapies, with only a single intended specified therapeutic function, engineered multifunctional NPs can be designed with capabilities of sensing and delivery of payloads of pharmacological activators, inhibitors, or genetic materials such as siRNA.^7–10^ The field of NP theranostics is currently dominated by applications in cancer medicine, but, in the past decade, a variety of NPs have also been developed to target different aspects of atherosclerosis.^11,12^ There are also many NPs originally designed for cancer that have high potential for atherosclerosis applications due to the extensive overlap in the biological mechanisms that govern tumours and atherosclerotic plaque development.^13,14^ These commonalities include risk factors, inflammation, and, most importantly, the role of macrophages.^15^

One exciting aspect of NP formulations is they can be engineered to specifically target certain key molecular elements or functions of a disease. Disease-specific molecular targets are particularly valuable as an aid to localize the delivery of NPs to pathological sites and to visualize high-risk or vulnerable foci within these tissues. In addition, specific targeting limits and/or avoids systemic and peripheral non-specific binding, thereby mitigating potential off-target effects. The targeting of intravascular atherosclerotic tissues using NPs can be achieved using a number of different approaches and combined with various imaging modalities (*Table 1*).

**Table 1:** Overview of macrophage-targeted theranostic NPs in preclinical models of atherosclerosis

Pub. year	Type of nanomaterial	Target ligand on NP	Imaging ligand	Imaging modality	Therapy	Experiment type	Cell type(s)/animal model(s)	Admin. route/frequency	Ref.
**Inorganic nanoparticles**									
**2006/2010**	Cross-linked coated iron oxide	Dextran	AF750	NIRF, MRI	PDT	In vitro/In vivo	RAW 264.7 murine macrophages *Apoe* ^−/−^ mice fed HCD	I/V	^16,17^
**2007**	Conjugated gold nanorods	Anti-CD11b antibody	Gold nanorods	Fluorescent	PTT	In vitro	RAW 264.7 murine macrophages	-	^18^
**2009**	Gold-coated iron oxide nanoclusters (nanoroses)	Dextran	Gold-coated iron oxide	NIRF, MRI	PTT	In vitro/In vivo	Macrophages, SMCs, ECs NZW rabbit (double-balloon injury + high-cholesterol, high-fat feeding)	I/V	^19^
**2013**	Coated gold nanorods	Poly-L-lysine	Gold nanorods	IVUS-IVPA	PTT	In vitro/ex vivo	J774A.1 murine macrophages. Ex vivo human coronary artery.	-	^20^
**2015**	Gold nanorods	(passive)*	Gold nanorods	CT	PTT	In vitro/in vivo	Ana-1 murine macrophages. Apoe−/− mice fed HCD with femoral artery cuff.	I/V	^21^
**2018**	Mannose-functionalized dendrimeric NPs	Mannose	FITC	Fluorescent	Liver-x-receptor (LXR) ligand	In vitro/in vivo	Mouse peritoneal macrophages and murine hepatocytes Ldlr−/− mice fed HCD.	I/V (weekly for 4 weeks)	^22^
**Carbon-based nanoparticles**									
**2012**	SWNTs	(passive)*	Cy5.5	NIRF	PTT	In vitro/in vivo	RAW 264.7 murine macrophages. FVB mice injected with STZ, fed HCD and carotid ligation.	I/V	^23^
**2020**	SWNTs	(passive)*	Cy5.5 or ^89^Zr	Fluorescence	SHP-1 inhibitor	In vitro/in vivo	RAW 264.7 murine macrophages, THP1, HCAEC, HCASMC. Apoe−/− mice fed HCD (chronic atherosclerosis) and angiotensin II infusions	I/V	^13^
**Lipid-based nanoparticles**									
**2010**	Liposomes	(passive)*	Gadolinium Rhodamine or Cy5.5	Fluorescence, MRI	Gluco-corticoids	In vivo	NZW rabbit model (double-balloon injury + high-cholesterol, high-fat feeding)	I/V	^24^
**2011**	siRNA carrying lipid NPs	(passive)*	AF647	Fluorescence	CCR2 siRNA	In vitro (siRNA screening)/In vivo	J774A.1 murine macrophages. Apoe−/− mice fed HCD	I/V	^25^
**2013**	PLGA-HDL hybrid NPs	Stearyl triphenyl phosphonium ligand	Quantum dots	Fluorescence	HDL	In vitro/in vivo	RAW 264.7 murine macrophages. Sprague–Dawley rats (biodistribution)	I/V	^26^
**2013**	Collagen-Specific Peptide Conjugated HDL NPs	Collagen-specific EP3533 peptides	Amphiphilic gadolinium chelates	MRI	HDL	In vivo	Reversa mice (model of atherosclerosis regression)	I/V	^27^
**2014/2015/2019**	Statin-loaded HDL-like NPs	(passive)*	Gadolinium or Cy5.5/DiO/DiR or ^89^Zr	Fluorescent, NIRF, MRI, PET	Simvastatin	In vitro/in vivo	J774A.1 murine macrophages21. Apoe−/− mice fed HCD20–22. Atherosclerotic NZW rabbit model (double-balloon injury and HCD feeding), Atherosclerotic porcine model.^28^	I/V low dose/long term (biweekly, 3 months) high dose/short term (4x, 1 week) treatment.^29^ 1 week + 8 weeks oral statin^30^	^28–30^
**2015**	PLGA (polylactic-coglycolic acid)−HDL-like NPs	(passive)*	Rhodamine or DiR	Fluorescent	HDL	In vitro/in vivo	J774A.1 murine macrophages (uptake compared with pancreatic ECs, murine aorta SMCs and HEPA hepatocytes). THP-1 human macrophages (chol. efflux). Apoe−/− mice fed HCD.	I/V	^31^
**2017**	HDL-like magnetic nanostructures	(passive)*	Fe_3_O_4_	MRI	HDL	In vitro	J774 murine macrophages (MRI and chol. efflux assays)	-	^32^
**2017**	HDL-like NPs	Hyaluronan	DiR	Fluorescent	Simvastatin	In vitro/in vivo	HUVEC human ECs/RAW 264.7 murine macrophages co-culture. Atherosclerotic NZW rabbit model (endothelial injury with high-fat diet)	I/V	^8^
**2018**	HDL-like NPs	(passive)*	DiO/DiR or ^89^Zr	Fluorescent, PET	Inhibitor of CD40-TRAF6 interaction	In vitro/In vivo	Murine BMDMs, RAW 264.7 murine macrophages. Apoe−/− mice fed HCD. Non-human primates.	I/V	^7^
**2020**	HDL-like synthetic hybrid NPs	Stearyl mannose and Stearyl triphenyl phosphonium	Iron oxide	MRI	HDL	In vitro/in vivo	RAW 264.7 murine macrophages (M2 polarized) + aortic SMCs. BALB/c mice.	I/V (lipid analysis + biodistribution after 24 h)	^33^
**2020**	Cargo-switching lipid-coated NPs	(passive)*	DiR	Fluorescent	Cholesterol dissolution, statin	In vitro/in vivo	RAW 264.7 + J774A.1 murine macrophages. Apoe−/− mice fed HCD, HCD vs. normal diet as regression study. Partial carotid ligation model.	I/V (biweekly, 4 weeks, HCD vs. normal diet (regression).	^34^
**2020**	siRNA carrying lipid NPs	S2P peptide	AF647	Fluorescent	CaMKIIγ siRNA	In vitro/in vivo	Murine BMDMs, Jurkat human T lymphocyte cells. BALB/c (pharmacokinetics/toxicity). Ldlr−/− mice fed HCD.	I/V	^35^
**2020**	Biomimetic macrophage membrane coated NPs	Macrophage membrane coating	Cy5 or Cy7.7	Fluorescent	Cytokine/chemokine sequestration, atorvastatin	In vitro/in vivo	HUVECs, RAW 264.7 murine macrophages. Apoe−/− mice fed HCD.	I/V (1 × dose, 6 h imaging, + weekly, 8 weeks)	^36^
**2022**	Ultrasound-responsive cyclodextrin NP	(passive)*	IR780 or PBCA microbubbles	NIRF, Ultrasound	Cyclodextrin	In vitro/in vivo	C57Bl/6, *Apoe*^−/−^ mice fed HCD.	I/V	^37^
**Other (hybrid/polymer) nanoparticles**									
**2013**	Protease-mediated, photosensitizer NPs (L-SR15)	(passive)*	Chlorin-e6	NIRF	PDT	In vivo	*Apoe−/−* mice fed HCD.	I/V (day 0, 7, and 14)	^38^
**2015**	Hyaluronic acid—polypyrrole NPs	Hyaluronic acid	Fluorescent doxorubicin	Fluorescent	Doxorubicin	In vitro	RAW 264.7 murine macrophages, VSMCs.	-	^39^
**2016**	Sugar-based amphiphilic macromolecule NPs	(passive)*	AF680	NIRF	α-Tocopherol or mucic acid (M12)	In vitro/in vivo	HMDMs, HCAECs, HCASMCs. Ex vivo human carotid arteries. Apoe−/− mice fed HCD.	I/V (day 0, 8, 17, and 25)	^40,41^
**2017**	Hyaluronan NPs	Hyaluronan	Cy7 or ^89^Zr	NIRF, PET	Hyaluronan	In vitro/in vivo	BMDMs. Apoe−/− mice fed HCD. NZW rabbit model (double-balloon injury + HCD feeding)	I/V (therapeutic: weekly for 12 weeks)	^42^
**2019**	Self-assembled, peptide-conjugated (CCTV) NPs	Anti-CCR2 peptide	Rhodamine or gadolinium or europium	Fluorescent, MRI	CCR2 blocking	In vitro/in vivo	Murine BMDMs, J774A.1 and RAW264.7 murine macrophages. Apoe−/− mice fed HCD.	I/V	^43^
**2019**	Fe-PFH-PLGA/chitosan-dextran sulphate NPs	Dextran-sulphate	Fe_3_O_4_	MRI	Ultrasound-induced phase transition	In vitro/ex vivo	RAW264.7 murine macrophages. Apoe−/− mice fed HCD aortas.	-	^44^

CT, computed tomography; HDL, high-density lipoprotein; IVUS-IVPA, intravascular-ultrasound photoacoustics imaging; MRI, magnetic resonance imaging; NP, nanoparticle; NIRF, near-infrared fluorescent imaging; PET, positron emission tomography; PDT, photodynamic therapy; PTT, photothermal therapy; SPECT, single-photon emission computerized tomography; SWNTs, single-walled carbon nanotubes; I/V, intravenous; HCD, high-cholesterol diet; *Apoe*^−/−^, Apolipoprotein E knockout; *Ldlr*^−/−^, low-density lipoprotein receptor knockout; NZW, New Zealand White; BMDM, bone marrow-derived macrophages; HMDM, human monocyte-derived macrophages; HCAECs, human coronary artery endothelial cells; HCASMCs, human coronary artery SMCs. *Indicates no active target ligand is present on the NP and macrophage uptake is achieved via general scavenging capacity of macrophages.

**Figure 1:**
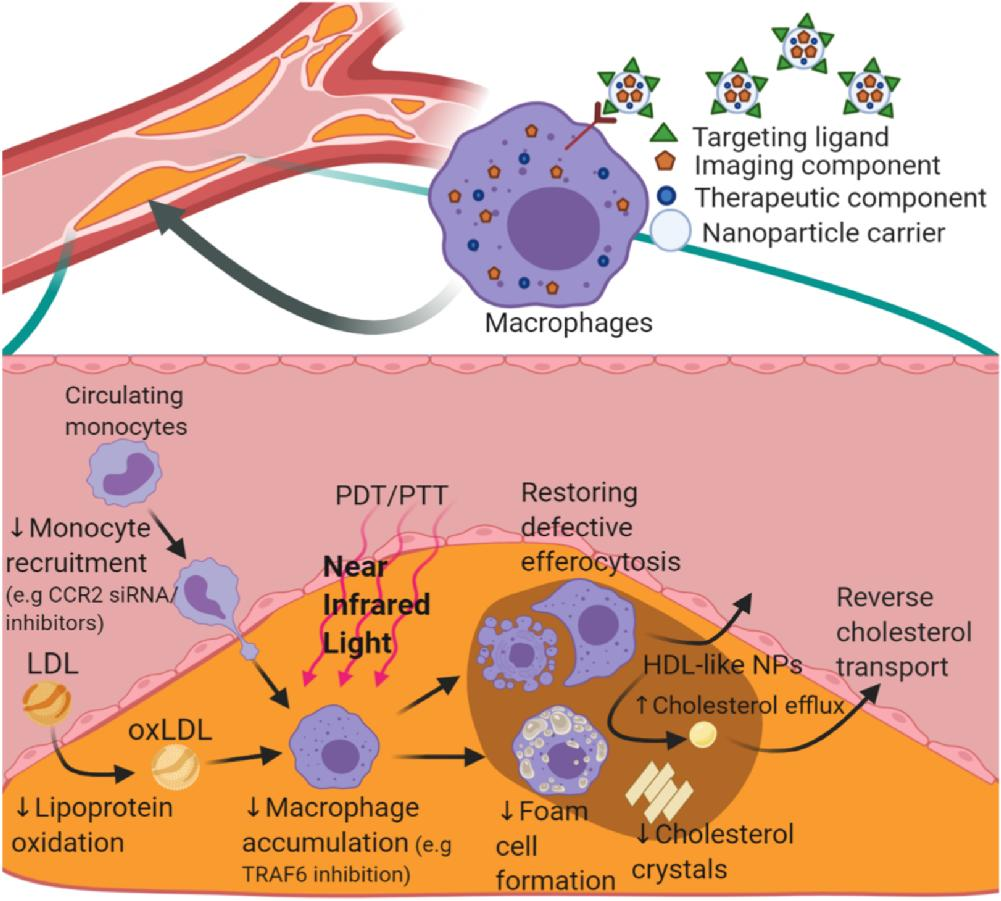
Initiation and progression of atherosclerosis—highlighting the role and origins of plaque macrophages. Atherosclerosis is initiated when LDL infiltrates into the subendothelial space where it undergoes oxidation to form oxLDL. Circulating bone marrow-derived monocytes are attracted to the site of oxLDL deposition by the activated endothelium releasing pro-inflammatory cytokines, attaching to the endothelial surface by adhesion molecules (ICAM/VCAM) and migrate across the endothelium. Within the intima, monocytes differentiate into macrophages to engulf the trapped oxLDL, leading to foam cell formation. Other potential sources of plaque macrophages include the trans-differentiation of SMCs to macrophage-like cells and additionally tissue-resident macrophages. Medial SMCs also migrate into the intima releasing collagen which contributes to the formation of the fibrous cap overlying the plaque. Once macrophages are unable to process the cholesterol from engulfed lipoproteins, they undergo apoptosis contributing to the formation of the necrotic core. Continued release of pro-inflammatory cytokines leads to further immune cell recruitment and infiltration, exacerbating atherosclerosis progression. This highlights the major contribution of plaque macrophages to atherosclerosis initiation and progression as well as the sources of these macrophages. LDL, low-density lipoprotein; oxLDL, oxidized LDL; VCAM1, vascular cell adhesion molecule-1; ICAM1, intercellular adhesion molecule-1; BM, bone marrow. Figure created with BioRender.com.

Endothelial cells and smooth muscle cells (SMCs) are important cell types in atherosclerosis; however, macrophages are highly prevalent in plaques and play critical roles in plaque development and instability. As a result, macrophages are well recognized as the best cellular target for the therapeutic modulation of atherosclerosis^45,46^ (*Figure 1*). By virtue of their essential role in the innate immune response, macrophages have excellent phagocytic properties, much greater than endothelial cells or SMCs, that can facilitate NP uptake.^47^ This feature can be especially beneficial for cytosolic or nuclear targets or delivery of bulky or poorly soluble therapies. NPs have been specifically functionalized to interact with different macrophage phenotypes, namely M1-like inflammatory macrophages and M2-like anti-inflammatory macrophages, using both passive and active mechanisms.^46^ Functional characteristics of macrophages such as efferocytosis can also be targeted.^48^

This review will describe the current status quo in the field of NPs that interact with macrophages and have therapeutic and/or specific imaging properties. In Section 2, we focus on strategies that target macrophages and the different functionalities of NPs applied in this context. In Section 3, we survey the NPs currently in development for other diseases that have high potential to be repurposed for application in atherosclerosis. The clinical translation of NPs, including current success stories and those with future potential, is discussed in Section 4. Section 5 outlines the current limitations in translating NPs to the clinic.

## 2. Theranostic NPs in atherosclerosis: the macrophage as a target

Many of the theranostic NPs designed for atherosclerosis are functionalized to target multiple aspects of macrophage biology and may also carry therapeutic cargo to provide additional benefit. Of these, the majority are lipoprotein-mimicking or micelle/liposome-based particles that can be loaded with therapeutic agents.

**Figure 2:**
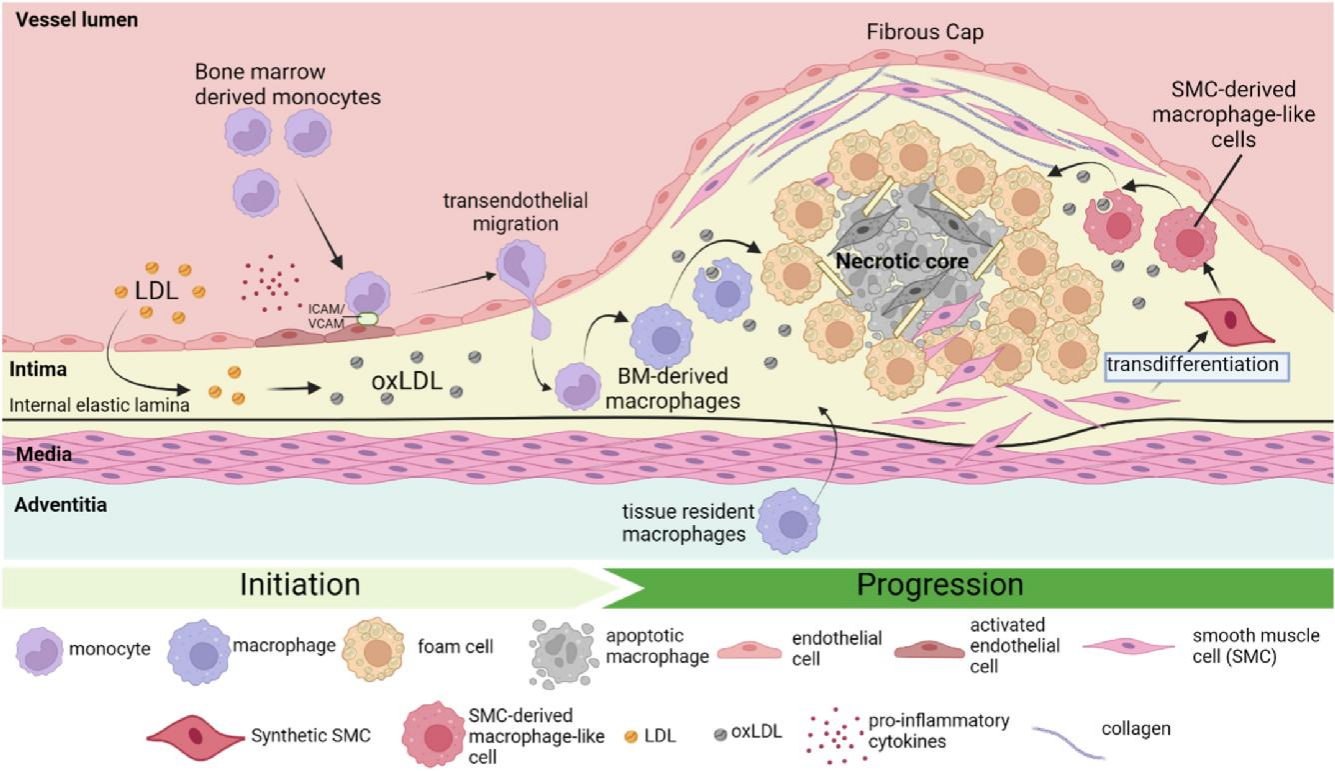
Using NPs to therapeutically target macrophages in atherosclerosis. Theranostic NPs typically consist of imaging/sensing and therapeutic components incorporated into a NP carrier. These NPs may also contain a ligand for targeting plaque macrophages and can therapeutically modulate a variety of pro-atherosclerotic mechanisms. Some of these NPs have utilized various therapeutic strategies to modulate the metabolism of low-density lipoprotein (LDL), such as decreasing monocyte recruitment via CCR2 inhibition using siRNA. These NPs prevent the accumulation of inflammatory cells and reduce LDL oxidation (oxLDL), which inhibits oxLDL uptake by macrophages. Molecular inhibition of mediators, such as TRAF6, also reduces macrophage accumulation. In addition, light-induced therapies such as photodynamic therapy (PDT) and photothermal therapy (PTT) can be used to cause macrophage ablation. Others have also attempted to restore defective efferocytosis to promote removal of apoptotic debris from the plaque. Promoting cholesterol efflux from plaque using high-density lipoprotein (HDL)-like NPs is another therapeutic strategy to decrease foam cell formation.

There are many targetable biological functions of macrophages for which an increasing number of NPs are being developed that modulate these functions. For example, there are NPs that reduce macrophage proliferation and inflammation and others that promote macrophage apoptosis and efferocytosis.^13,24,28–30,36^ NPs that modify macrophage foam cell-related processes have been developed including those that promote reverse cholesterol transport or inhibit LDL oxidation to reduce foam cell formation^40^ (*Figure* *2*). Several cell surface markers on macrophages have been utilized in NP designs to facilitate their macrophage targeting. This includes targeting to markers that designate macrophage sub-types with different functionalities. For M1-like pro-inflammatory macrophages, MHC-II, CD80, and CD86 markers are used, whereas for anti-inflammatory M2-like macrophages, CD36, CD206, and CD163 markers are utilized.^46^ This section will provide relevant background on macrophages in relation to macrophage targeting and expand on key examples of NPs that target macrophages and modify their functions, many of which are in the preclinical phase of development.

### 2.1 NP fate and the role of macrophages

As NPs enter the bloodstream following intravenous administration, they are coated with a biomolecular corona (typically consisting of opsonin proteins) which is integral to their resultant biodistribution, pharmacokinetics, and ability to take up NPs.^49–51^ Classically, the uptake of NPs is primarily mediated at the frontline by macrophages, which leads to clearance by the mononuclear phagocyte system via the liver and spleen. There are several strategies that help to slow clearance via this pathway such as coatings of polyethylene glycol. These coatings prevent mononuclear cell recognition and increase circulation time, which in turn increases the number of NPs that reach the macrophages recruited into plaques.^49,52^

NPs are able to reach the target site of the atherosclerotic plaque and then be taken up by macrophages when delivered intravenously. Plaque uptake can be via a passive mechanism by virtue of the fact that the endothelium that overlays the plaque has a higher permeability^53^ with evidence suggesting that when plaques stabilize this reduces the accumulation of the NPs at the plaque site.^54,55^ In more established plaques, additional entry opportunities exist via plaque neovessels that protrude into plaques and are also fragile.^56^

### 2.2 Plaque macrophage sources and phenotypes in atherosclerosis

#### 2.2.1 Sources of macrophages

Macrophages are found throughout a host of different organs and tissues including adipose tissue, liver, lungs, and intestinal tract. Due to the chronic inflammatory nature of atherosclerotic plaques, a high proportion of plaques are comprised of macrophages. These macrophages are derived from different origins and can have different expression profiles that can affect the likelihood of progression or regression of the plaque and their ability to be recognized by different NPs.^57^

The traditional view is that the majority of plaque-associated macrophages are derived from circulating monocytes, recruited from the bloodstream, and originating from the bone marrow. The accumulation of macrophages in plaque can also be driven locally by proliferation.^58^ Vascular resident macrophages, derived from yolk-sac progenitor cells, make further contributions to the macrophage content of the plaque.^59^ There are observations of tissue resident macrophages that are sourced from the vessel wall, residing in the adventitia, and that increase in number in hyperlipidaemic mouse models of atherosclerosis.^60^

Another important source of plaque macrophages is from the transdifferentiation of vascular SMCs to macrophage-like cells,^61,62^ with the observation that plaque foam cells can originate from SMC-derived macrophage-like cells. In high-lipid atherogenic conditions, SMCs lose their contractile phenotype and convert to a proliferative and migratory synthetic phenotype that allows for transdifferentiation into macrophages. Cholesterol loading of SMCs *in vitro* is found to downregulate expression of SMC-specific markers including α-actin and calponin but conversely upregulates macrophage markers such as CD68 and ABC-A1.^62^ It has been revealed that at least 50% of foam cell plaque macrophages are SMC derived.^63–65^ Interestingly, SMC-derived macrophages are not as efficient at either phagocytosis or efferocytosis when compared with monocyte-derived macrophages.^64^ Expression profiling of intimal SMC-derived macrophages found lower levels of cholesterol transporter ABCA1, suggesting reduced cholesterol efflux capacity and enhanced foam cell formation.^63,65^ Much remains unknown regarding the regulation of SMC transdifferentiation into macrophages. There is evidence that the pluripotency gene Kruppel-like factor 4 has a key role^66^ along with miR143/145 that regulates myocardin, a key transcription factor in SMC differentiation.^64^ More recently, lipase A that encodes lysosomal acid lipase (LAL) was reported to be downregulated in SMC-derived foam cells when compared with macrophage foam cells *in vitro.* LAL hydrolyses cholesterol ester and increases cytoplasmic lipid droplet formation, with SMC-derived foam cells having accumulation of lipid in the lysosomal compartment, suggesting another defining feature of SMC transdifferentiation and foam cell formation.^67^

Targeting NPs to plaque macrophages with differing origins might be beneficial, although limiting targeting to a specific source may not achieve the same level of therapeutic or diagnostic benefit. Rather, NPs that are recognized by the majority of macrophages irrespective of their source are likely to be of the most value. For most NPs, the inherent phagocytic nature of macrophages ensures their uptake, as long as they have compatible size or charge properties. The lower phagocytic ability of SMC-derived plaque macrophages is something to be considered, and NPs designed to target key markers of these such as CD68 (also present on all other macrophages) may enhance recognition and efficacy. An interesting example of nanotechnology exploiting the plasticity of SMCs was peptide amphiphile micelle NPs designed to target SMCs via the CCR2 receptor (using a CCL2 peptide) and delivering miR-145 to promote a more contractile phenotype. This strategy was able to inhibit plaque growth in atherosclerotic *Apoe*^−/−^ mice at both early and later stages.^68^

#### 2.2.2 Macrophage phenotypes

One of the more intriguing aspects of macrophages is the plasticity and heterogeneity of macrophage populations within the atherosclerotic plaque. As macrophages are highly adaptive cells, they can respond to the local microenvironment and molecular cues to modify their characteristics appropriately. Despite the existence of highly heterogeneous macrophage populations, a broad classification of macrophages has involved dividing macrophages into two major sub-types, M1 and M2.

Pro-inflammatory M1 macrophages are differentiated in response to toll-like receptor (TLR) and interferon-γ signalling following exposure to pro-inflammatory stimuli including lipopolysaccharide (LPS), lipoproteins, and pathogen-associated molecular patterns. M1 macrophages secrete pro-inflammatory factors such as interleukin-1β (IL-1β) and tumour necrosis factor-α (TNF-α). In addition, M1 macrophages secrete reactive oxygen species (ROS) and nitric oxide NO that initiates and sustains inflammation.^69^ In contrast, M2 macrophages exhibit primarily anti-inflammatory properties and are differentiated in response to Th2-type cytokines IL-13 and IL-4. M2 macrophages secrete anti-inflammatory factors such as IL-10 and transforming growth factor-β that favour inflammatory resolution.^70^ In the context of the M1/M2 polarization paradigm, the predominate macrophage subtype found in atherosclerotic plaque is the M1 subtype. In atherosclerotic plaque, M1 macrophages are found in unstable, rupture-prone regions and M2 macrophages in the more stable regions and adventitia.^57^

The vast heterogeneity of atherosclerotic plaque macrophages makes it challenging to target a specific macrophage phenotype with a NP and impart the maximum benefit. In atherosclerosis, all macrophages, even the M2 macrophages, contribute in deleterious ways to plaque development. NP technologies are most likely to have therapeutic and diagnostic efficacy when recognized by the spectrum of macrophage phenotypes.

### 2.3 Targeting macrophage mediated inflammation

Inhibition of inflammation has been proved to be a viable strategy to reduce recurrent cardiovascular events.^71^ The CANTOS trial tested canakinumab, a monoclonal antibody against the pro-inflammatory cytokine IL-1β. Canakinumab significantly reduced high-sensitivity C-reactive protein levels from baseline and lowered the incidence of recurrent cardiovascular events, when compared with placebo.^71^ One drawback to systemic IL-1β inhibition was the finding of a higher rate of death from infections.^71^ This highlights that improved targeting of inflammation towards atherosclerotic plaques using NPs^6,72^ could be a highly effective strategy to reduce the off-target effects of systemically delivered immunosuppressing therapies.^24,28,29,73^

Statins are widely used oral cholesterol-lowering medications that inhibit 3-hydroxy-3-methylglutaryl coenzyme A reductase to reduce cholesterol synthesis. Statins also exhibit anti-inflammatory properties at high doses. This anti-inflammatory effect can be enhanced further when a high dose is specifically targeted to the plaque site, thereby avoiding potential off-target effects from systemic high-dose delivery.^29^ One example of the success of this approach is reconstituted high-density lipoprotein (rHDL)-based NPs loaded with simvastatin (S-HDL). S-HDL improved the bioavailability of the statin cargo, with an increased accumulation of S-HDL in plaque macrophages.^29^ S-HDL reduced plaque inflammation and macrophage proliferation that exceeded free statin therapy, in atherosclerosis-prone apolipoprotein (*Apo*)*e*^−/−^ mice.^29,30^ S-HDL was tracked using a combination of fluorescence, by incorporating DiO in the hydrophobic core or Cy5.5 in the lipid monolayer, and magnetic resonance imaging (MRI) via the addition of gadolinium.^29,30^ S-HDL also successfully reduced inflammation in large preclinical models of atherosclerosis including the rabbit and pig,^28^ indicating the high potential for clinical translation.

A similar strategy has been used with liposome NPs loaded with encapsulated prednisolone phosphate (L-PLPs), a glucocorticoid known for its anti-inflammatory properties.^24^ These L-PLPs were also labelled with the MRI-detectable paramagnetic lipid gadolinium (Gd-DTPA-BSA) and the fluorescently labelled lipid rhodamine-PE (0.2%). The L-PLPs were retained in the atherosclerotic plaque of rabbits and found to reduce plaque inflammation. Drug delivery was also able to be monitored using MRI. L-PLPs make use of non-specific targeting, relying on passive uptake at the plaque site rather than the use of targeted ligands. Although the preclinical applications of L-PLPs were promising, a clinical trial of L-PLP particles found no significant anti-inflammatory effects, despite detection of L-PLPs in plaque macrophages.^73^

Recently, Gao *et al*.^36^ reported the development of biomimetic NPs that utilize a macrophage membrane coating to reduce clearance by the reticuloendothelial system and to facilitate targeting towards areas of inflammation, such as atherosclerotic plaques. The authors also hypothesized that the macrophage membrane coating could sequester pro-inflammatory cytokines by the presence of multiple membrane receptors (e.g. CD36, CCR2, TNFR2) that bind pro-inflammatory cytokines/chemokines and inhibit inflammation. In addition, these NPs incorporated a ROS-responsive core that enables the specific release of the payload, in this case, atorvastatin, upon exposure to oxidation at the plaque site. Overall, the combined effect of potential sequestration of pro-inflammatory cytokines released by macrophages with a pharmacological treatment reduced atherosclerotic plaque inflammation and area in *Apoe*^−/−^ mice.^36^

### 2.4 Inhibiting monocyte migration and recruitment, and macrophage accumulation

During atherosclerosis progression, circulating monocytes are recruited to the vessel wall, where they differentiate into macrophages and contribute to plaque development. An interesting approach by Lameijer *et al.*^7^ capitalized on this pathway by utilizing an rHDL-based NP containing a small molecule inhibitor of tumour necrosis factor receptor-associated factor 6 (TRAF6i). The rationale behind using this inhibitor was to intercept CD40-TRAF6 interactions in myeloid cells, which in turn inhibits monocyte recruitment to atherosclerotic plaques and plaque development.^74^ TRAF6i-rHDL NPs were taken up by monocytes and macrophages, but not lymphocytes, and indeed led to a decrease in plaque macrophage content and plaque inflammation.^7^ By incorporating a fluorescent dye and ^89^Zr, TRAF6i-rHDL biodistribution was observed via fluorescence and positron emission tomography (PET) imaging in *Apoe*^−/−^ mice and non-human primates. Overall, by targeting monocyte migration it demonstrated that an inhibitor of CD40-TRAF6 signalling could be delivered at the plaque site using rHDL NPs and prevent atherosclerosis.

Another way to target monocyte entry into plaques is by intercepting chemokine/chemokine receptor interactions. CCL2 is a potent pro-inflammatory chemokine that facilitates the recruitment of inflammatory monocytes to the plaque via interaction with its receptor CCR2.^75^ Drawing on this idea, Leuschner *et al.*^25^ utilized a fluorescently labelled lipid NP that delivered an siRNA to knockdown CCR2 in mice. This NP reduced the numbers of plaque monocytes and macrophages and was detectable by fluorescence imaging. Another interesting approach involving CCR2 targeting was the design of a peptide-conjugated nanoparticle (CCTV) that incorporated a peptide antagonist of CCR2.^43^ These NPs were able to target inflammatory monocytes expressing CCR2 (CCR2^hi^Ly6C^hi^) and cause down-regulation of inflammatory gene expression in these cells in the blood and atherosclerotic plaque. CCTV particles were also able to be chelated to gadolinium to enable MRI of atherosclerosis in *Apoe*^−/−^ mice.^43^

Hyaluronan is a polymer found in the extracellular matrix that is involved in several biological processes, including cell adhesion, proliferation, and migration. It has predominantly been utilized as an agent that assists with reducing NP uptake by the liver. It has, however, also been used to modulate inflammatory responses, acting as a therapeutic element of the NP structure. Hyaluronan-coated nanoparticles (HA-NPs) are able to target various receptors expressed on plaque macrophages, with hyaluronan-binding receptors including CD44, ICAM-1, LYVE-1, and TLR-4 that are involved in both monocyte adhesion and inflammation. Beldman *et al.*^42^ showed that HA-NPs were specifically taken up by plaque-associated macrophages and displayed anti-inflammatory effects by reducing macrophage accumulation in plaques. ^89^Zr-labelled HLA-NPs were also be used for PET imaging of atherosclerosis in rabbits and to detect their uptake in atherosclerotic regions in the aortas of mice.^42^

Overall, these studies provide evidence that NPs designed to inhibit monocyte migration and recruitment and macrophage accumulation are effective therapeutic approaches for preventing and detecting atherosclerosis.

### 2.5 Reduction of lipoprotein oxidation/foam cell formation

The uptake of oxidized LDL into macrophages is mediated via the scavenger receptor CD36, a key driver of atherosclerosis. Lewis *et al*.^40,41^ used a NP-based strategy to target this pathway which involved the development of sugar-derived amphiphilic macromolecule NPs.^40,41^ The sugar-derived macromolecule components of these NPs mimic the charge and hydrophobicity of oxidized LDL and therefore compete for binding with CD36. Furthermore, upon interaction with CD36, antioxidant α-tocopherol cargo is released from the NP locally to the macrophage. The overall effect of this NP design was a reduction in the uptake of oxidized lipids in macrophages via CD36, which could also be tracked via the inclusion of fluorescent labelling (AF-680) for imaging.^41^

The promotion of macrophage cholesterol efflux is another anti-atherogenic strategy that reduces accumulation of intracellular cholesterol and suppresses foam cell formation. A specifically designed polymeric NP with mannose-functionalized dendrimeric NPs has been utilized to target and increase the activity of the liver-X-receptor (LXR) in plaque macrophages. LXR is a transcription factor that upregulates the cholesterol transporters ABCA1 and ABCG1 and therefore cholesterol efflux.^22^ LXR ligands were specifically delivered to plaque macrophages in atherosclerotic LDL receptor (*Ldlr*)^−/−^ mice using this approach, as detected by fluorescent imaging that was enabled by conjugation of a FITC fluorophore. This approach resulted in an increase in cholesterol efflux and a reduction in plaque progression, necrosis, and inflammation.^22^

As well as being an endogenous liposomal NP, HDL also promotes cholesterol efflux. The capacity of HDL to facilitate cholesterol efflux is retained even when the internal core components are modified to contain other cargo.^31^ HDL-like magnetic nanostructures (HDL-MNS) consisting of an Fe_3_O_4_ core have been developed that promote cholesterol efflux in a comparable manner to native HDL, in parallel to improving MRI contrast in cultured macrophages.^32^ HDL-MNS particles have not, however, been investigated in an *in vivo* model of atherosclerosis, but have potential for this application due to their interactions with macrophages and HDL-like properties.^32^

During atherogenesis, the accumulation of both cholesterol and macrophages in plaques results in macrophage foam cells and cholesterol crystals that are highly pro-inflammatory. Cargo-switching nanoparticles (CSNPs), designed by Kim *et al.*,^34^ consist of a lipid-coated cyclodextrin–statin complex. Upon exposure of this complex to cholesterol crystals, the cyclodextrin dissolved the cholesterol crystals and the CSNP complex released the statin drug payload at the plaque site.^34^ This strategy increased the specificity of statin delivery and reduced plaque inflammation and cholesterol accumulation in macrophage foam cells. Mehta *et al.*^37^ also tested cyclodextrin-loaded NPs in an air-trapped polybutylcyanoacrylate NP design. IR780 dye was also incorporated for detection via both near-infrared (NIR) and ultrasound imaging modalities. Although cyclodextrin dissolves cholesterol crystals, it also has a low retention time, requiring high doses to achieve effective therapeutic effects. With this design, these cyclodextrin NPs enhanced the anti-atherosclerotic properties of cyclodextrin in *Apoe*^−/−^ mice. Upon exposure to ultrasound, cyclodextrin was released, increasing its uptake into macrophages.

### 2.6 Restoring defective macrophage efferocytosis

Efferocytosis is an important process performed by macrophages that removes apoptotic cellular debris. This process can, however, become defective with the progression of atherosclerosis. An approach by Flores *et al.*^13^ utilized single-wall carbon nanotubes (SWNTs) to target plaque macrophages and promote efferocytosis. SWNTs are preferentially taken up by the Ly-6C^hi^ subset of activated monocytes that are known to subsequently differentiate into plaque macrophages.^76^ As efferocytosis is dysfunctional in atherosclerosis, the authors designed a SWNT-based NP that included an inhibitor of Src homology 2 domain-containing phosphatase-1 (SHP-1) which is downstream of the receptor CD47. CD47 is a receptor that binds to ‘don’t eat me’ ligands and is upregulated in atherosclerotic plaque, including unstable plaque. These NPs inhibited the CD47 signalling cascade in inflammatory monocytes/macrophages and restored efferocytosis in atherosclerotic plaque. This study found that SWNT NPs accumulated in plaque, improved the phagocytic ability of macrophages, and reduced plaque burden in *Apoe*^−/−^ mice.^13^

Tao *et al.*^35^ investigated a NP-based strategy that incorporated an siRNA that targeted Ca^2+^/calmodulin-dependent kinase γ (CAMKIIγ). CAMKII γ is a calcium-activated kinase that has previously been shown to be activated in advanced plaque and promote the development of unstable necrotic atherosclerotic plaques with thin fibrous caps.^35^ This mechanism is driven by the suppression of MerTK, a key receptor involved in efferocytosis. The NPs were designed to have a polylactic-coglycolic acid (PLGA) core loaded with CAMKIIγ siRNA and cationic lipid complexes. These particles also incorporated a S2P peptide to target the particles to the stabilin receptor on macrophages. The authors demonstrated that these NPs lowered CAMKIIγ and increased MerTK expression in the macrophages of atherosclerotic plaques. An increase in efferocytosis, a smaller necrotic core area, and increased fibrous cap thickness were also observed. This indicates that CAMKIIγ-silencing NPs directly modified macrophage activity and increased the stability of atherosclerotic plaque by increasing efferocytosis.

### 2.7 Inducing macrophage ablation via light-inducible therapies

As well as targeting macrophage functionality, direct ablation of macrophages has been investigated as a potential strategy for preventing atherosclerosis. The main techniques used to induce ablation are photodynamic and photothermal therapies.^77^

#### 2.7.1 Photodynamic therapy

Photodynamic therapy involves the use of a photosensitizer which, upon exposure to a specific wavelength of light, causes cells to produce ROS (radicals and singlet oxygen). The ROS, in turn, induce cytotoxicity and ablation of cells that have taken up the photosensitizer.^77^ Photosensitizers also commonly carry fluorescent properties, enabling direct imaging.

There are a couple of examples of theranostic NPs that use photodynamic therapy for macrophage ablation in atherosclerosis. Shon *et al.*^38^ utilized photodynamic therapy with ‘L-SR15’ NPs. These were composed of multiple chlorin e6 (Ce6) molecules conjugated to a biodegradable poly-l-lysine backbone. This enabled aggregation and self-quenching so that the phototoxicity and fluorescence properties of the Ce6 were switched off.^38,78^ The photodynamic therapy and imaging properties of Ce6 were therefore only activated in the presence of cathepsin-B, a macrophage-associated, plaque-destabilizing protease. In the carotid plaque of mice, L-SR15 NPs were shown to reduce plaque macrophage content following photodynamic therapy (stimulation with light), attenuate cathepsin-B activity, and enable near-infrared fluorescence (NIRF) imaging of Ce6 that localized to the plaque region.^38^

A cross-linked dextran-coated iron oxide (CLIO) theranostic NP has also been utilized that enables photodynamic therapy.^16^ The polysaccharide dextran coating of these NPs facilitates uptake by phagocytic macrophages via lectin-receptor-mediated endocytosis.^79^ The CLIO particles were modified to include a chlorine-based photosensitizer that, when illuminated with a 650-nm laser, was able to specifically induce macrophage ablation. In addition, fluorescent AF-750 was incorporated to enable the detection of plaque macrophages.^16^

#### 2.7.2 Photothermal therapy

Photothermal therapy uses photoabsorbers that induce hyperthermia upon exposure to light to induce death in target cells. Primarily based on gold nanorod and gold NPs, photothermal therapy typically results in the ablation of macrophages by inducing cellular hyperthermia. Some examples of photothermal therapy used for atherosclerosis have shown some, albeit limited, preclinical efficacy in reducing plaque macrophages.^80^ As an example, Qin *et al.*^21^ used gold NPs to apply photothermal therapy for inflammatory macrophage ablation. These NPs enabled photothermal therapy both in cultured macrophages and in plaque macrophages in atherosclerotic *Apoe*^−/−^ mice upon irradiation with an 808-nm NIR laser. The gold NPs also enabled imaging at the plaque site by microCT.

Other photothermal therapy approaches have included silica-coated nanorods which allow for a combination of photothermal therapy with plaque imaging using intravascular ultrasound and photoacoustics (IVUS/IVPA). These nanorods were designed for simultaneous targeting of macrophages for photothermal therapy and could also monitor the temperature during heating of the NPs.^20^

SWNTs have also been utilized for photothermal therapy as they are able to induce thermal ablation upon exposure to light in the NIR range. SWNT Cy5.5-labelled NPs were taken up by cultured macrophages and, upon irradiation with NIR, induced macrophage apoptosis. In a carotid ligation model of atherosclerosis, SWNT Cy5.5-labelled NPs accumulated in plaques and caused macrophage apoptosis upon exposure to NIR light. In addition, these particles could be detected using the Cy5.5 tag for *in vivo* fluorescence imaging.^23^

Despite the potential benefits of having light-induced therapeutic strategies incorporated into NP platforms, some translational issues persist. The photosensitizers or photoabsorbers require specific wavelengths of light that do not have the necessary penetration depth to enable non-invasive treatment of atherosclerosis in deeper tissues, for example, in the coronary arteries. To overcome this, these technologies would ideally need to be used in conjunction with an intravascular endoscope-based light source, which carries its own limitations and risks for a patient.

**Figure 3:**
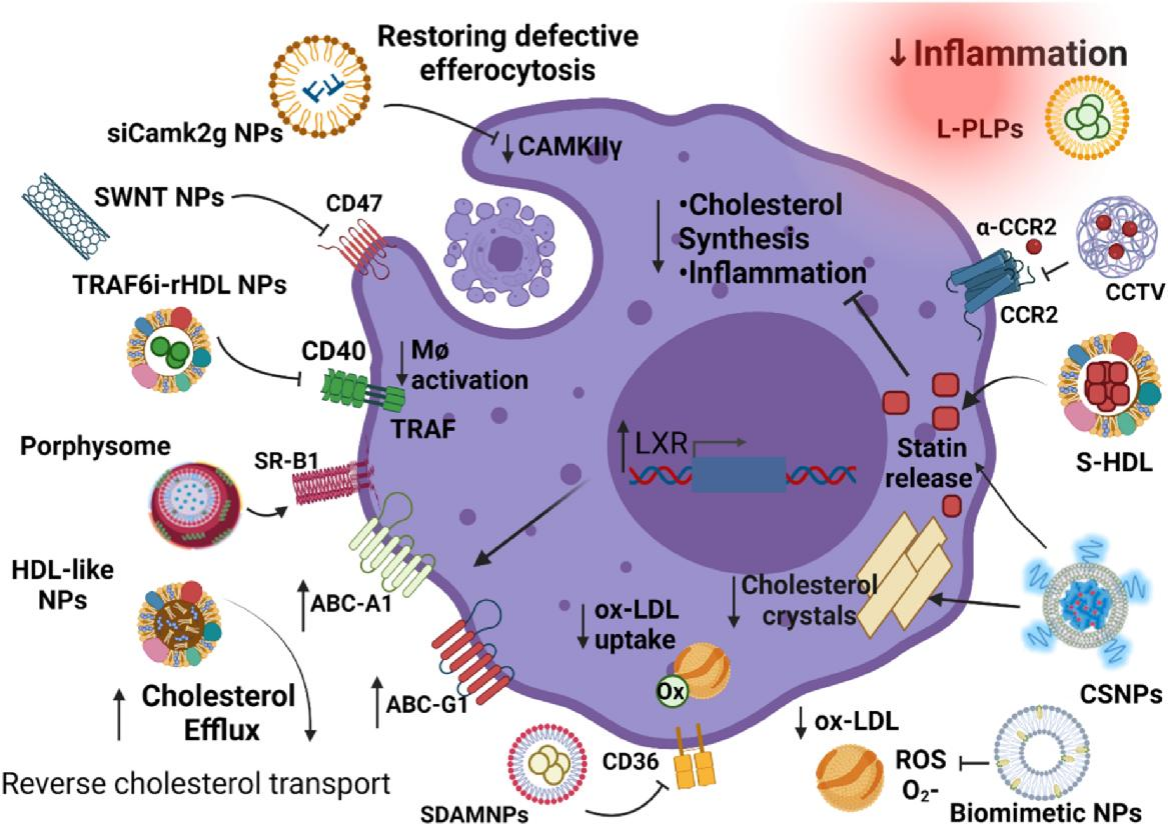
Macrophage functions targeted by NPs that reduce atherosclerosis. Theranostic NPs have been designed to target a host of macrophage functions and elicit beneficial effects including: (i) anti-inflammatory effects using statin release from statin-HDL (sHDL) and cargo-switching NPs (CSNPs) as well via antagonism of CCR2 using a peptide in a peptide-conjugated NP (CCTV) and through liposome-based NPs loaded with anti-inflammatory prednisolone phosphate (L-PLPs); (ii) inhibiting the uptake of oxidized LDL (ox-LDL) using biomimetic NPs that release their payload when they come in contact with ROS and also through direct inhibition of the scavenger receptor CD36 using sugar-derived amphophilic macromolecule NPs (SDAMNPs) that release anti-oxidant α-tocopherol upon interaction with CD36; (iii) enhancing cholesterol efflux using NPs that activate LXR which increases the expression of the cholesterol transporters ABCA1 and ABCG1, and porphysomes that efflux cholesterol via the scavenger receptor SR-B1; (iv) restoring defective efferocytosis using SWNT that inhibit CD47 and through NPs that cause siRNA-mediated inhibition of Ca2+/calmodulin-dependent kinase γ (siCAMk2 g NPs); and (v) finally, through inhibition of monocyte activation using a small molecule inhibitor of TNF receptor-associated factor 6 (TRAF6) loaded inside HDL-like NPs (TRAF6i-rHDL).

In summary, there is an ever-increasing number of promising NPs with theranostic capabilities for application in atherosclerosis using a range of ingenious strategies that allow targeting to plaque macrophages (*Figure 3*). Despite the importance of clinically translating these nanoscale technologies, this currently remains limited (Section 4). An increased understanding of their properties in biological systems is necessary to facilitate these important next translational steps and to further understand their immense theranostic potential.

## 3. Repurposing NPs for applications in atherosclerosis

NPs have been studied extensively in a host of different pathologies, including cancer, infection, autoimmunity, and inflammation.^81,82^ A number of these NP platforms exhibit properties that lend them excellent efficacy in atherosclerotic applications. Here, we discuss examples of such platforms that are highly promising for application in atherosclerosis.

### 3.1 Theranostic NPs used in cancer applications

Cancer and atherosclerosis share several common mechanisms such as oxidative stress, inflammation, angiogenesis, and defective efferocytosis.^15,83^ In both disease conditions, many of these mechanisms are associated with macrophage function. Through their ability to directly target disease sites, NPs present as an extremely promising solution for both cancer and atherosclerosis to overcome the deleterious off-target effects caused by systemic delivery of therapies. A number of inorganic and carbon-based NPs that target different types of macrophages (M1 and M2) have been reported to exhibit anti-cancer effects.^84^ Pro-inflammatory, M1-like macrophage-targeting NPs have been used to treat the negative effects of inflammation in cancer.^84^ M2-like macrophages that secrete anti-inflammatory cytokines are also prospective delivery vehicles for NP-based cancer therapies due to their phagocytic abilties.^84^ Similar mechanisms controlled by M1, M2, and other types of macrophages (M (Hb), Mox, and M4) operate in the development, progression, and regression of atherosclerotic plaque.^45,57^ In addition, lipid-based nanoparticle (LNP) platforms exist that have been successfully applied in cancer models and specifically target macrophage, which have high potential for translational applications in atherosclerosis.^85–88^

#### 3.1.1 Porphyrin–lipid NPs

Porphyrin–lipid liposomes or ‘porphysomes’ are NPs with a liposome-like structure with porphyrin–lipid conjugate (minimum 5%) in their outer layer.^89,90^ These porphysomes and other porphyrin–lipid NPs have been extensively studied for applications in targeted tumour imaging and photodynamic therapies.^89,91–94^ The inclusion of porphyrin–lipid is an especially useful feature of porphysomes. Porphyrin has intrinsic multi-modal imaging capabilities with NIR fluorescence properties and is a natural chelator of transition metals, enabling radiolabelling with ^64^Cu for PET imaging or manganese for MRI.^90–92,95^ Within the porphyrin–lipid NP sub-family, a number of different compositions have been developed with properties that lend them to be highly efficient theranostic NPs for atherosclerosis.^89,96–98^ For example, porphyrin–lipid NPs have been designed to target the scavenger receptor SR-BI which is highly expressed on cancer cells.^99^ This was achieved by incorporating the apoA-I mimetic peptide R4F to form HDL-mimetic porphylipoproteins (PLPs). PLPs were found to track to tumours in preclinical cancer models, detected using both fluorescence and PET imaging modalities, and enabled specific photodynamic therapy at the tumour site.^90^ Interestingly, SR-B1 is also highly expressed on atherosclerotic plaque macrophages where it mediates both the uptake and efflux of cholesterol, as well as anti-inflammatory pathways.^100^ The SR-B1-targeting capabilities of PLPs therefore suggest they will target plaque macrophages, promote cholesterol efflux, and exhibit anti-inflammatory effects.

Porphysomes have also been modified with other targeting ligands such as folate, which enables targeting to the folate receptor that is highly expressed on activated macrophages.^101^ Folate-porphysomes successfully tracked to activated macrophage infiltrates in a murine MI model measured using both non-invasive fluorescent and PET imaging modalities.^101^ Another feature of porphysomes and other porphyrin–lipid NPs is that their core can be loaded with therapeutic cargo (including RNAi therapies) or other imaging agents, further adding to their theranostic capabilities.^90^ Overall, the multiple properties and imaging capabilities of porphyrin–LNPs strongly suggest their potential as excellent theranostic agents for atherosclerosis.^101^

#### 3.1.2 Lanthanide-based up-/down-conversion NPs

Lanthanide-based up-/down-conversion NPs (UCNPs and DCNPs) have also been tested in cancer models and offer a unique strategy for imaging multiple cellular targets or markers simultaneously in tissues.^102–104^ Unlike most fluorescent sensors, these NPs are excited by NIR radiations with lower energy (longer wavelength) rather than conventional visible radiations and can selectively emit fluorescence at wavelengths across infrared, visible, and ultraviolet. This method serves to eliminate background signals and enables high tissue penetration depths.^105^ A range of lanthanide-doped NPs have been engineered with different luminescence lifetimes providing the opportunity for *in vivo* multiplexing of several colocalized targets. Fan *et al*.^104^ reported that lifetime-engineered NIR-II (imaging in the second NIR window) NPs create a tuneable lifetime range spanning three orders of magnitude, but only using a single emission band. Due to these unique properties, they only require a single laser and detector and therefore a cheaper overall system. Furthermore, imaging readouts are not impacted by the scattering of different wavelengths, ensuring inter-channel comparisons are real. These NPs were successfully used for *in vivo* multiplex imaging of live mice in which multiple breast tumour biomarkers and their distribution patterns within the tumours were non-invasively visualized.^104^ Recently, Yang *et al*.^106^ demonstrated that thulium-based cubic-phase downshifting NIR-II NPs doped with Er^3+^ or Ho^3+^ facilitated non-invasive real-time dynamic multiplexed imaging of cerebrovascular vasomotion activity. This included single-cell vision of neutrophil extravasation into subcutaneous tissues in an ischaemic stroke model. With the heterogeneity of atherosclerotic plaque development, the ability to simultaneously detect a host of relevant plaque markers and in real-time dynamically, lanthanide-based UCNPs and DCNPs have the potential to vastly improve the accuracy of identifying features of, for example, vulnerable high-risk plaques or the movements of key cell types such as macrophages in and out of plaques.

### 3.2 NPs for detection of inflammation and infection

Cytokines play a significant role in inflammation which underlies a host of diseases. Cytokine sensing platforms are therefore transferable across a range of pathological conditions, especially atherosclerosis in which the levels of inflammation align with the progression and vulnerability of plaques.^107,108^ Liu *et al*.^109^ reported a NP-based affinity sensor, the ‘OnCELISA’, which is able to specifically detect the cell from which inflammatory cytokine IL-6 was secreted and the amount in macrophages *in vitro* (BV2 microglia). This applicability of OnCELISA was then used to detect IL-6-expressing cells using flow cytometry in atherosclerotic plaque-containing mouse aortae digested into single-cell suspensions.^109^ This demonstrates a clear application in atherosclerosis in which the OnCELISA technology could be used to detect key atherosclerotic inflammatory proteins specifically released from macrophages for the improved diagnosis of atherosclerosis.

NPs have also been tested in different macrophage phenotypes *in vitro* to demonstrate anti-inflammatory applications in the context of infection and sepsis. For example, Foit and Thaxton^110^ developed HDL-like NPs functionalized to inhibit the Toll-like receptor 4 (TLR4)-dependent inflammatory response to LPS. These NPs suppressed TLR4 signalling, causing a significant reduction in inflammatory cytokine secretion in human THP-1 cells and human peripheral blood mononuclear cells that were exposed to LPS derived from multiple bacterial species.^110^ TLR4 is important mediator of the inflammasome response that plays a key role in atherosclerosis.^111^ Blockade of the TLR4 signalling pathway using these HDL-like NP platforms therefore has the potential for repurposing for the early detection and/or mitigation of inflammatory ‘hot spots’ in atherosclerotic plaque.

Recently, Khalid *et al*.^112^ demonstrated that diamond-based NPs could be used to detect changes in temperature down to a resolution of 0.1°C non-invasively in a wide physiologically relevant range. Negatively charged nitrogen vacancy colour centres in nanodiamond particles exhibit optically detected magnetic resonance and act as nanoscale thermometers.^113,114^ The application for this was in wound healing, in which the nanodiamonds were woven into electrospun silk fibroin scaffolds and placed on wounds for the detection of temperature changes, a key indicator of infection and inflammation.^112^ Temperature sensing using diamond-based NPs^112^ also presents as a potential strategy to detect vascular inflammation and the high levels of inflammation associated with unstable plaque that have local inflammation-induced elevations in temperature. For example, previous studies have found there is thermal heterogeneity in regions of carotid and coronary atherosclerotic plaque.^115,116^ In carotid enterectomy samples, this heterogeneity represented temperature variability in the range of 0.4–2.2°C in 37% of plaques.^115,117^ Thermal heterogeneity was found to be more pronounced in the coronary plaque of individuals with unstable angina or previous acute MI than those with normal or stable angina.^116,118^ This temperature heterogeneity has also been correlated with plaque composition such as macrophage density.^119^ Interestingly, microwave radiometry has been explored for use as a non-invasive method to detect some the thermal heterogeneity of plaque, with an accuracy of ±0.2°C (at 32–38°C).^117^ Taken together, the thermal heterogeneity associated with atherosclerotic plaque and vascular inflammation suggests nanodiamonds have potential to be utilized as a nanoscale thermometers to sense local changes in plaque temperature as a marker of stability.^112^

## 4. Clinical translation of theranostic NPs in cardiovascular disease

Despite the rapidly expanding literature on the testing of NPs in preclinical models of atherosclerosis, currently Clinicaltrials.gov shows very few clinical trials related to atherosclerosis and NPs (*Table 2*). Although this platform does not cover all clinical trials, the very low number indicates it is considerably challenging to make the clinical translation step for NPs targeted at atherosclerosis. So far, the NANOM-FIM trial^120^ tested silica–gold NPs with plasmonic photothermal therapy (PPTT) properties which were delivered either via an arterial patch or via an intravenous catheter to patients with coronary artery disease after which they were irradiated with a NIR laser. Although a stark reduction occurred in fibrous tissue, which could indicate plaque destabilization, the NP-treated plaques using both application techniques proved superior to stenting in reducing the local plaque volume. Another clinical trial has also been performed with a liposomal NP encapsulating prednisolone phosphate (LN-PLP).^73^ Here, analysis of plaque macrophages from patients with iliofemoral atherosclerosis, who were scheduled for endarterectomy, showed that most plaque macrophages internalized the NPs following venous infusion. Although a promising result, in a general cardiovascular disease patient cohort there was no apparent effect of these NPs on arterial wall permeability, or inflammatory status as assessed by PET imaging.^73^

**Table 2:** Examples of NPs in cardiovascular disease clinical trials

Year	Study Name	Nanomaterial	Target ligand on NP	Therapeutic/diagnostic agent	Study design	Phase	Objectives/outcomes	Patient cohort	Primary outcome measures	Ref./Clinical trial number
**2015**	NANOM-FIM, Plasmonic Nanophotothermal Therapy of Atherosclerosis	Silica-gold NP	Bioengineered arterial patch or targeted microbubble/stem cell delivery	Plasmonic photothermal therapy (PPTT)	Multicentre, randomized, double-blind, observational study	-	Completed. Safety and feasibility of Silica-gold NP- mediated PPTT of plaque in patients with CAD. Reduced TAV and risk of cardiovascular death. 5-year follow-up revealed high level safety, fewer MACE and less target lesion revascularisation. Cytotoxicity from Silica gold NPs in blood.	CAD with angiographic SYNTAX score ≤22. 180 patients (final)	TAV by quantitative coronary angiography, intravascular ultrasound, MACE-free survival	NCT01270139^120–122^
**2015**	SILENCE, silencing inflammatory activity by injecting nanocort in patients at risk of atherosclerotic disease	liposomal NP with encapsulated prednisolone phosphate (LN-PLP)	Liposomal NP	Prednisolone (anti-inflam, therapeutic)	Randomized, double-blind, placebo-controlled study	I/II	Completed. Accumulation of LN-PLP in plaque macrophages but no reduction in plaque inflammation.	30 patients	FDG PET-CT	NCT01601106^73^
**2019**	PAC-MAN, treatment of patients with atherosclerotic disease with paclitaxel associated with LDL-like nanoparticles	Lipid-nanoemulsion (LDE)—paclitaxel NP (Paclitaxel-LDE)	LDL-like coating (targeting LDLR on macrophages)	Paclitaxel (therapeutic)	Prospective, randomized, double-blind, placebo-controlled study	II/III	Safety and efficacy of Paclitaxel-LDE in patients with stable CAD. Ongoing	Stable CAD. 40 patients	Low-attenuation plaque volume (LAPV) by coronary or aortic CTA	NCT04148833
**2020**	Treatment of patients with atherosclerotic disease with methotrexate associated with LDL-like nanoparticles	Lipid-nanoemulsion (LDE)—methotrexate NP (MTX-LDE)	LDL-like coating (targeting LDLR on macrophages)	Methotrexate (anti-inflam, therapeutic)	Prospective, randomized, double-blind, placebo-controlled study	II/III	Safety and efficacy of MTX-LDE in patients with stable CAD. Ongoing	Stable CAD. 40 patients	Coronary and aortic CT angiography	NCT04616872
**2020**	ORION	Inclisiran PCSK9 LNP	GalNac-Lipid NP (to liver)	PCSK9 siRNA (therapeutic)	Randomized, double-blind, placebo-controlled study	III	Completed. Approved. 50% reduction in LDL cholesterol when administered every 6 months.	ORION-10: 1561 patients; ORION-11: 1617 patients. Patients with CAD (ORION-10) and with CAD or ASCVD disease risk equivalent (ORION-11).	Percent change in LDL cholesterol compared with placebo.	NCT03399370 NCT03400800^123^
**2022**	VT-1001, A study of VERVE-101 in patients with FH and CVD	VERVE-101 lipid nanoparticle	Lipid NP (to liver)	PCSK9 CRISPR base editing (therapeutic)	Single-ascending dose, non-randomized study	I	Ongoing. Safety of VERVE-101	HeFH ASCVD, and Uncontrolled Hyper-cholesterolaemia 44 patients (estimated).	Incidence of TEAEs, SAEs and AESIs	NCT05398029
**2023**	VT-10201, A study of VERVE-102 in patients with FH or premature CAD	VERVE-102 lipid nanoparticle	GalNac-lipid NP (to liver)	PCSK9 CRISPR base editing (therapeutic)	Single-ascending dose, non-randomized study	I	Ongoing. Safety of VERVE-102	HeFH or Premature CAD. 36 patients (estimated).	Incidence of TEAEs and SAEs	NCT06164730

CAD, coronary artery disease; CRISPR, clustered regularly interspaced short palindromic repeats; PCSK9, proprotein convertase subtilisin/kexin type 9; ASCVD, atherosclerotic cardiovascular disease; TAV, total atheroma volume; USPIOs, ultrasmall superparamagnetic iron oxides; HeFH, heterozygous familial hypercholesterolaemia; TEAE, treatment-emergent adverse events; SAEs, serious adverse events; AESIs, adverse events of special interest; FH, familial hypercholesterolaemia.

Recent phase II/III clinical trials have also been initiated that are testing methotrexate-loaded (NCT04616872) and paclitaxel-loaded (NCT04148833) LDL-like NPs for their safety and efficacy in patients with stable coronary disease. Methotrexate is an anti-inflammatory agent previously tested in the CIRT trial at a low dose in patients with a history of MI and coronary artery disease. In this trial, low-dose methotrexate did not reduce cardiovascular events compared with placebo.^124^ To improve targeting to plaques and to maximize the anti-inflammatory effects without unwanted systemic side effects, NPs have now been designed. A lipid-nanoemulsion coating (LDE) has been developed, resembling LDL, that can acquire ApoE proteins upon contact with plasma, enabling uptake by LDL receptor-expressing cells such as inflammatory plaque macrophages.^85,87^ Prior testing in rabbits found LDE–methotrexate NPs, formulated with a lipophilic methotrexate derivative, reduced plaque area by 65%.^85^ Another LDL-like NP loaded with anti-proliferative agent paclitaxel (LDE-paclitaxel) caused a similar 60% reduction in plaque area in rabbits.^86^ A pilot study in humans found LDE-paclitaxel was well tolerated in patients with aortic atherosclerosis.^88^

Overall, these trials demonstrate promising results and potential, but also highlight that the clinical application of NPs for the treatment and diagnosis of atherosclerosis is still in its infancy and major efforts are needed to create a clinical breakthrough. There are, however, a number of exciting new LNPs that modulate lipid metabolism and lower LDL cholesterol which have demonstrated outstanding efficacy. They do not, however, target plaque macrophages or have imaging capabilities, yet highlight the potential of NP strategies to reduce cardiovascular disease. Packaged with genetic materials (e.g. siRNAs, CRISPR/Cas9 mRNA), the molecular targets of this new generation of LNPs include proprotein convertase subtilisin/kexin type 9 (PCSK9) and angiopoietin-like protein 3 (ANGPTL3). They are designed to lower LDL cholesterol levels by inhibiting gene expression in the liver. Some have been FDA-approved, whilst others are in various stages of clinical trial development. A number of these nanotherapeutics take advantage of N-acetyl galactosamine (GalNAc)-LNP encapsulation that facilitates uptake via the asialoglycoprotein receptor which is highly expressed on hepatocytes.^125^ This has advantages over other LNPs that target the LDL receptor as the LDL receptor is expressed at low levels in some individuals, limiting uptake. One LNP utilizing GalNAc is inclisiran, a PCSK9 siRNA GalNAc-LNP that has demonstrated lasting reductions in LDL cholesterol for up to 6 months and is currently approved for use in patients with hypercholesterolaemia.^123^

There has also been recent exciting exploration of base-editing CRISPR/Cas9 technology in LNPs to permanently alter the expression of lipid metabolism genes in the liver. These studies are currently at either the preclinical stage or in early clinical testing. Verve therapeutics developed VERVE-101, which utilizes base-editing CRISPR technology that targets PCSK9. VERVE-101 is a therapeutic LNP formulation for hypercholesterolaemia that provides excellent durable and potent inhibition of PCSK9 and long-lasting reductions in LDL cholesterol of over one year.^126,127^ This has been demonstrated in mice and non-human primates and has moved towards phase I trial testing (NCT05398029). A second formulation, VERVE-102 is also in development that utilizes the GalNAc-LNP liver delivery system (NCT06164730).

ANGLTL3 is another therapeutic target for atherosclerotic CVD acting as an endogenous inhibitor of lipoprotein lipase, the main enzyme involved in the hydrolysis of triglyceride rich lipoproteins.^128^ A GalNAc-LNP targeting ANGPTL3 with CRISPR has demonstrated a reduction in ANGPTL3 levels 6 months post-dosing in non-human primates, and the GalNAc formulation was favourable for specific base editing in the liver.^129^

Whilst these liver-targeted lipid-modulating LNPs are highly promising future therapeutics for atherosclerosis, many patients require more than lipid lowering alone to prevent secondary events. They are also unable to enhance plaque detection beyond current imaging strategies. NPs that target alternate therapies to plaques via macrophages are a vital pursuit that has the potential to shift the dial on improving disease detection and secondary event prevention.

## 5. Limitations in the clinical translation of nanotechnologies for cardiovascular disease

A range of polymeric NPs, polymeric micelles, liposomal NPs, nanocrystal formulations, protein, and inorganic NPs have been successful in obtaining approval by regulatory agencies for different pathological conditions.^130^ Of the total number of nanomedicines on the market or in the clinical trial stage, 53% are for cancer applications with only 3% for cardiovascular diseases.^131^ This demonstrates that there are specific challenges in the translatability of cardiovascular disease-targeted nanomedicines. Accurate targeting to plaque is one prominent challenge. Culprit plaques reside within blood vessels in the heart, ruling out localized topical or subcutaneous delivery approaches. The parenteral route of delivery, commonly intravenous injection, is therefore required that raises the risk of off-target effects by exposing the NPs to the entire circulatory system. This is also associated with increased cost to administer these nanomedicines to patients due to the need for medical facilities and trained personnel.^132^

The biodistribution and clearance of NPs depend on the size of the particle, the polarity, circulation dynamics, pharmacokinetics, and receptor affinity/binding^50,133^ . Appropriate biodistribution of NPs is critical in clinical applications as they determine the efficacy as well as acute or chronic side effects. This is also important to ensure that NP treatments reach the targeted tissue efficiently. This is particularly crucial considering the distribution of macrophages throughout different tissues and organs, this requires specific targeting of NPs to plaque macrophages. It is important to keep in mind that there are also resident macrophages in the liver (Kupffer cells) and that the liver is able to sequester a large proportion of NPs that are introduced into the bloodstream.^134^ This could also be a reason why inclisiran and other liver-targeted Gal-Nac-LNPs that alter lipid metabolism have seen more success in clinical translation, with targeting NPs for uptake in hepatocytes a more efficient process than targeting atherosclerotic plaque. Additionally, this highlights the need to ensure that NPs are designed to specifically target plaque macrophages and to reduce the non-specific uptake by other tissue resident macrophages. Efforts to better characterize the activity and expression profiles of plaque macrophages might also reveal novel ways to improve specific targeting of NPs to atherosclerotic plaque.

Many NPs also carry unique toxicity concerns when compared with conventional medicines. There are several classes of nanomaterials that have different considerations for biocompatibility and toxicity when introduced to a biological system, which can often be unpredictable.^135^ This introduction of NPs within a biological system exposes them to active biomolecules, forming a protein corona on the surface of the NPs. This corona is integral to influencing the toxicity of NPs and how they subsequently interact *in vivo*.^135^ The chemical composition of NPs is also an important factor that influences toxicity as well as size and charge characteristics.^132^ Liver toxicity can also be a factor, and if NPs are non-degradable, then they can accumulate in the liver and can lead to longer-term changes in gene expression that contributes to chronic toxicity.^134^ Therefore, even if a nanomaterial demonstrates efficacy, there is the added factor of nanotoxicity that must be considered and tested during the preclinical stage. For example, despite efficacy demonstrated by of the NANOM-FIM trial,^121^ the use of the gold–silica NPs observed cytotoxicity, reported in a long-term sub-analysis of the 5 year follow up.^122^ This also demonstrates the need to additionally examine the long-term safety outcomes of NP-based therapies.

Another consideration is that even though NPs have shown safety and efficacy in other pathologies, there may be potential for unintended effects on atherosclerosis. Atherosclerosis is driven by chronic inflammation in the vessel wall. Therefore, even if a NP formulation is shown to localize to plaque, if the nanomaterial stimulates inflammatory responses, this may exacerbate atherosclerosis progression. An example includes prednisolone-loaded liposomes (discussed in Section 4) that accumulated in plaque macrophages in patients but did not provide any anti-inflammatory or therapeutic effects and in patients with advanced atherosclerosis seemed to increase arterial wall inflammation.^73^ Further investigation in *Ldlr*^−/−^ mice revealed that the liposomal formulation of the anti-inflammatory prednisolone had the paradoxical effect of increasing inflammatory cell infiltration in plaques and advanced the stage of atherosclerosis in these mice. This was shown to most likely be caused by suspected lipotoxic effects of the liposomal NPs in macrophages located in a lipid-rich milieu.^136^ This exacerbation of atherosclerosis progression has also been induced by silica NPs (siNPs) that elevated serum triglycerides and LDL-C levels. These siNPs also activated endoplasmic reticulum stress and macrophage infiltration within the plaque in *Apoe^−/−^* mice.^137^ Overall, this illustrates the importance of detailed investigations into the effect of NPs on immune responses and whether this might negate the desired therapeutic effects on atherosclerosis.

Another limitation of NP translation is the scalability of the production and batch-to-batch variability, which needs to be considered during the design phase.^138^ This ability to scale up production is important as NPs tend to agglomerate at higher concentrations that can affect activity and subsequent functional characterization.^139^ Agglomeration is also dictated by the physical properties of NPs including zeta potential (particle surface charge), size, and composition as well as interaction with serum. This alters the effective size leading to recognition by the MPS and rapid clearance by the liver and spleen.^132^ However, some like Binderup *et al*.^28^ have carefully considered the need to have scalability in NP design, switching to a microfluidics platform to produce volumes of S-HDL NPs required for larger animal (rabbit and porcine) preclinical stages. The quality of the materials needed to formulate the NP is also an important consideration. These determine whether the final product can be safely manufactured and, for example, is pyrogen-free or can be sterilized.^132^

## 6. Conclusions and future perspectives

Atherosclerosis is a complex pathological process, in which molecular imaging and targeted therapeutic delivery are critical for effective diagnosis, treatment, and monitoring of disease progression. Engineered NPs of different types, composition, and biocompatibility profiles, along with their inherent and modifiable properties, make them versatile platforms for theranostic applications in atherosclerosis. Whilst there is currently little evidence supporting a role for nanoscale technologies in primary prevention, there is immense potential for a clinical impact in the secondary prevention of atherosclerosis-related complications by facilitating early detection, identification of vulnerable plaque, and targeted therapeutic interventions.

Macrophages play a significant role in the development, progression, and destabilization of plaques and are also able to interact with NPs via general phagocytic mechanisms or specific/targeted uptake. Macrophage-directed theranostics are therefore a logical and highly promising strategy for atherosclerosis. The combination of the versatile chemical properties of NPs and biological function of macrophages has led to the emergence of many imaging and therapeutic applications. Of the nanoscale theranostics that are targeted towards macrophages, the majority are currently in the preclinical phase, with many only being tested *in vitro* and very few in (large) animal models of atherosclerosis. As with other diseases, many challenges exist when looking to translate preclinical applications of NPs to clinical successes in the cardiovascular space.

In this review, we have drawn attention to macrophages as the key cellular target mediating the effectiveness of NPs against atherosclerosis. As with all clinical applications of NPs, it will be essential to control the biodistribution and pharmacokinetics and to identify the potential off-target or toxic effects. The transferable knowledge and technology from similar applications in other pathological conditions such as cancer and infection should be incorporated in designing or repurposing such theranostic applications for atherosclerosis. Moreover, a more integrated interdisciplinary approach to set up studies that involve chemists, pharmacologists, physicists, biologists, and clinicians will be vital to move this field forward and tackle the complex biological and clinical questions associated with atherosclerosis.

## Author’s Contributions

V.N., A.K.V., A.H., and C.A.B. conceived and drafted the manuscript. J.T.M.T., J.V., P.J.P., M.R.H., B.C.G., Y.J., E.G., and G.Z. critically reviewed the manuscript and provided important intellectual content.

## Data Availability

There are no new data associated with this manuscript.
